# Udder pathogens and their resistance to antimicrobial agents in dairy cows in Estonia

**DOI:** 10.1186/1751-0147-53-4

**Published:** 2011-02-08

**Authors:** Piret Kalmus, Birgit Aasmäe, Age Kärssin, Toomas Orro, Kalle Kask

**Affiliations:** 1 Department of Therapy, Institute of Veterinary Medicine and Animal Science, Estonian University of Life Sciences, Tartu, 51014, Estonia; 2 Department of Environment and Animal Health, Institute of Veterinary Medicine and Animal Science, Estonian University of Life Sciences, Tartu, 51014, Estonia; 3 Estonian Veterinary and Food Laboratory Tartu 51014, Estonia

## Abstract

**Background:**

The goal of this study was to estimate the distribution of udder pathogens and their antibiotic resistance in Estonia during the years 2007-2009.

**Methods:**

The bacteriological findings reported in this study originate from quarter milk samples collected from cows on Estonian dairy farms that had clinical or subclinical mastitis. The samples were submitted by local veterinarians to the Estonian Veterinary and Food Laboratory during 2007-2009. Milk samples were examined by conventional bacteriology. *In vitro *antimicrobial susceptibility testing was performed with the disc diffusion test. Logistic regression with a random herd effect to control for clustering was used for statistical analysis.

**Results:**

During the study period, 3058 clinical mastitis samples from 190 farms and 5146 subclinical mastitis samples from 274 farms were investigated. Positive results were found in 57% of the samples (4680 out of 8204), and the proportion did not differ according to year (p > 0.05). The proportion of bacteriologically negative samples was 22.3% and that of mixed growth was 20.6%. *Streptococcus uberis *(*Str. uberis*) was the bacterium isolated most frequently (18.4%) from cases of clinical mastitis, followed by *Escherichia coli *(*E. coli*) (15.9%) and *Streptococcus agalactiae *(*Str. agalactiae*) (11.9%). The bacteria that caused subclinical mastitis were mainly *Staphylococcus aureus *(*S. aureus*) (20%) and coagulase-negative staphylococci (CNS) (15.4%). The probability of isolating *S. aureus *from milk samples was significantly higher on farms that had fewer than 30 cows, when compared with farms that had more than 100 cows (p < 0.005). A significantly higher risk of *Str. agalactiae *infection was found on farms with more than 600 cows (p = 0.034) compared with smaller farms. The proportion of *S. aureus *and CNS isolates that were resistant to penicillin was 61.4% and 38.5%, respectively. Among the *E. coli *isolates, ampicillin, streptomycin and tetracycline resistance were observed in 24.3%, 15.6% and 13.5%, respectively.

**Conclusions:**

This study showed that the main pathogens associated with clinical mastitis were *Str. uberis *and *E. coli*. Subclinical mastitis was caused mainly by *S. aureus *and CNS. The number of *S. aureus *and *Str. agalactiae *isolates depended on herd size. Antimicrobial resistance was highly prevalent, especially penicillin resistance in *S. aureus *and CNS.

## Background

Bovine mastitis is the most common disease in dairy cows worldwide, and antimicrobial therapy is the primary tool for the treatment of mastitis. The prevalence of mastitis pathogens and their antimicrobial resistance have been investigated in numerous studies around the world. The main pathogens that cause subclinical mastitis are coagulase-negative staphylococci (CNS), *Corynebacterium bovis *(*C. bovis*) and *Staphylococcus aureus *(*S. aureus*) [1-5]. Coliforms, *Streptococcus uberis *(*Str. uberis*) and *S. aureus *are the pathogens isolated most frequently from clinical mastitis samples [6-8]. *Streptococcus agalactiae *(*Str. agalactiae*) has been largely eradicated from herds in Europe [3], but in studies from the United States, 7.7% and 13.1% of samples contained *Str. agalactiae *[9,10].

Several methods, such as disc diffusion, agar dilution, broth dilution and broth microdilution are suitable for *in vitro *antimicrobial susceptibility testing. Depending on the study design and the methodology used, the antimicrobial susceptibility of udder pathogens varies greatly between studies. For example, studies from France and the UK have reported a high prevalence of penicillin-resistant *S. aureus *(36.2%, 56%) [11,12], whereas a low percentage of resistant isolates (4-9%) were found in the Netherlands and Norway [13,14]. The streptococci that cause mastitis are susceptible to β-lactam antibiotics; however, resistance to macrolides and lincosamides is notable [13,15]. *In vitro *resistance of *E. coli *to different antimicrobials has been reported to be low [13,14,16,17].

National studies of mastitis prevalence provide important information through the monitoring of national udder health status, and they enable national guidelines to be developed for the prudent use of antibiotics in each country [18]. During recent decades, only broad-spectrum antibiotics have been used for the treatment of clinical mastitis in Estonia. For example, in the years 2006-2009, 15 different combinations of antibiotics were available for use in 18 intramammary preparations that were authorised by the Estonian State Medical Agency [19]. Given that a large overview of udder pathogens and their antibiotic resistance has not been performed in Estonia, the goal of this study was to estimate the distribution of udder pathogens and their antibiotic resistance during the years 2007-2009 in Estonia.

## Methods

### Sample collection

Milk samples were submitted to the Estonian Veterinary and Food Laboratory during the period 2007-2009. Quarter milk samples were collected from cows on Estonian dairy farms by local veterinarians or farmers. Clinical mastitis was diagnosed when visible abnormalities of udder (swelling) were detected or milk from a quarter had abnormal viscosity (watery, thicker than normal), colour (yellow, blood-tinged) or consistency (flakes or clots) [20]. Normal milk appearance, together with a positive California Mastitis Test result (score greater than 1), was used to make a diagnosis of subclinical mastitis.

The samples were sent to the laboratory either for isolation of the clinical mastitis pathogen and determination of its antimicrobial susceptibility or to determine the reason for an increased somatic cell count.

### Laboratory analysis

Bacterial species were identified using accredited methodology based on the National Mastitis Council [21] standards. From each sample, 0.01 ml of milk was cultured on blood-esculin agar and incubated for 48 h at 37°C. The plates were examined after 24 and 48 h of incubation. A minimum of five colonies of the same type of bacterium was recorded as bacteriologically positive, and growth of more than two types of bacterial colonies was categorised as mixed growth. No bacterial growth was recorded when fewer than five colony-forming units were detected during 48 h of incubation.

Once they had been isolated and identified, pure cultures of udder pathogens were tested for antibacterial susceptibility with the disc diffusion assay on Mueller-Hinton agar. Testing was performed according to the recommendation of the Clinical and Laboratory Standards Institute (CLSI) document M31-A2 in the years 2007-2008 and M31-A3 in 2009 [22,23]. Quality control strains, *S. aureus *ATCC^® ^25923, *E. coli *ATCC^® ^25922, *Pseudomonas aeruginosa *ATCC^® ^27853 and *Streptococcus pneumoniae *ATCC^® ^49619, were included with each batch of isolates tested. The antimicrobial susceptibility of Gram-positive bacteria was tested with penicillin, ampicillin, cephalothin, clindamycin, erythromycin, gentamycin, trimethoprim/sulfa and tetracycline. The antimicrobial susceptibility of Gram-negative bacteria was tested with ampicillin, gentamycin, trimethoprim/sulfa, tetracycline, enrofloxacin, streptomycin, neomycin and cefaperazone. The list of antibiotics in susceptibility testing may vary, different veteriarians preferred different set of antibiotics in order to find accurate treatment after getting the laboratory test results.

The criteria for the interpretation of zone diameter used in this study are described in Table 1 .

**Table 1:** Zone diameter intepretive criteria

Disc content in μg	*Staphylococcus *spp.			*Streptococcus *spp.			***Enterococcus *spp**.			*Enterobacteriaceae *spp.		
	S	I	R	S	I	R	S	I	R	S	I	R
Ampicillin 10 μg	≥ 29	-	≤28	≥ 26	19-25	≤18	≥ 17	-	≤16	≥ 17	15-16	≤14
Penicillin 10 μg	≥ 29	-	≥ 29	≥24	-	-	≥15	-	≤14	-	-	-
Cephalothin 30 μg				≥		≤	-	-	-			
Cefaperazone 75 μg	-	-	-	-	-	-	-	-	-	≥21	16-20	≤15
Clindamycin 2 μg	≥ 21	15-20	≥ 14	≥19	16-18	≤15	-	-	-	-	-	-
Erythromycin 15 μg	≥ 23	14-22	≥ 14	≥21	16-20	≤15	-	-	-	-	-	-
Gentamycin 10 μg	≥ 12	13-14	≥ 15	≥12	13-14	15≤	≥10	7-9	≤6	≥ 12	13-14	≥ 15
Tetracycline 30 μg	≥ 19	15-18	≥ 14	≥23	19-22	≤18	≥19	15-18	≤14	≥ 19	15-18	≥ 14
Enrofloxacin 5 μg										≥ 20	15-19	≤14
Trimethoprim/sulfa 1,25/23,75 μg	≥ 16	11-15	≥ 10	≥16	11-15	≤10	≥16	11-15	≤10	≥ 16	11-15	≥ 10

### Data analysis

The farm, herd size and year were recorded and categorised before statistical analysis. A logistic regression model with a random herd effect for the control of clustering was used for all of the analyses in this study. Odds ratios (OR) with 95% confidence intervals (95% CI) were calculated. Statistical significance was set at p ≤ 0.005.

The influence of milk samples with mixed growth or no bacterial growth on the occurrence of clinical or subclinical mastitis was assessed. Potential interactions (no growth or mixed growth × year) were assessed in the logistic regression model. The effects of herd size and year on the pathogens that caused clinical and subclinical mastitis were analysed. These analyses were conducted using Stata 10.2 [24].

## Results

### Isolation of mastitis pathogens

During the study period, 3058 clinical mastitis samples from 190 farms and 5146 subclinical mastitis samples from 274 farms were investigated (Table 2).

**Table 2:** Distribution of milk samples according to herd size

	Clinical mastitis				Subclinical mastitis			
**Farm size category**	**Farms**	**%**	**Samples**	**%**	**Farms**	**%**	**Samples**	**%**

1 (1-30 cows)	54	28.4	98	3.2	41	15	86	1.7
2 (31-99 cows)	35	18.4	149	4.9	51	18.6	268	5.2
3 (100-299 cows)	40	21.1	378	12.4	53	19.3	541	10.5
4 (300-599 cows)	44	23.2	1472	48.1	80	29.2	2426	47.1
5 (> 600 cows)	17	8.9	961	31.4	49	17.9	1825	35.5
Total	190	100	3058	100	274	100	5146	100

Positive results were found in 57% of the samples (4680 out of 8204), and this proportion did not differ according to year (p > 0.05). The proportion of bacteriologically negative samples was 22.3% and that of mixed growth 20.6%. There was a significantly higher chance (OR = 1.15, 95% CI = 1.01, 1.33, p = 0.042) of finding bacteriologically negative samples in presence of subclinical mastitis (n = 1317, 25.6%) in comparison with clinical mastitis (n = 554, 16.8%). The probability of obtaining mixed growth from milk samples was also significantly higher (OR = 2.2, 95% CI = 1.9, 2.6, p < 0.001) if subclinical mastitis was found. The distribution of bacterial species isolated from samples from cows with clinical and subclinical mastitis is shown in Table 3. Among the bacteriologically positive (n = 2016) clinical mastitis samples, *Str. uberis *was the bacterium isolated most frequently (n = 371; 18.4% of the positive samples), followed by *E. coli *(n = 321; 15.9%) and *Str. agalactiae *(n = 293; 11.9%). *S. aureus *(n = 532; 20%) and CNS (n = 411; 15.4%) were the bacteria isolated most commonly from milk in cases of subclinical mastitis, followed by *Corynebacterium *spp. (n = 395; 14.8%).

**Table 3:** Distribution of bacterial species isolated from clinical and subclinical mastitis samples in 2007-2009

	Clinical mastitis			Subclinical mastitis		
**Bacteria**	**2007** **(n = 598)**	**2008** **(n = 692)**	**2009** **(n = 726)**	**2007** **(n = 939)**	**2008** **(n = 1063)**	**2009** **(n = 661)**

*S. aureus*	11.7	11.7	11.7	19.2	22.8	16.6
CNS	4.8	7.1	8.5	16.1	13.6	17.4
CPS*	3.8	3.3	1.6	4.6	2.8	5.1
*Str. agalactiae*	9.0	11.3	14.7	13.6	9.0	10.7
*Str. dysgalactiae*	8.0	7.8	7.2	3.6	4.0	5.6
*Str. uberis*	16.1	21.8	17.1	10.2	12.3	12.9
*Str*. spp	3.2	3.3	1.9	1.2	2.0	2.7
*Lactococcus lactis*	10.9	3.9	5.7	8.9	8.2	3.9
*E. coli*	14.4	16.6	16.5	1.6	2.0	3.8
*Klebsiella *spp.	7.0	1.3	2.3	0.7	0.6	0.9
*Enterococcus *spp.	1.3	2.3	1.1	1.5	2.8	4.2
*Corynebacterium *spp.	2.2	2.6	5.0	16.5	17.3	8.5
*A. pyogenes*	2.2	3.8	3.6	0.1	0.6	0.6
*Pseudomonas *spp.	1	0.3	0.3	0	0	0.6
*Proteus *spp.	0.2	0	0.2	0.4	0.1	0.6
Yeast	2.3	2	1.6	1.5	1.6	5.6
Other	1.8	0.9	1	0.3	0.3	0.3
Total	100%	100%	100%	100%	100%	100%

* CPS: coagulase-positive staphylococci (other than *S. aureus*)

The probability of isolating *S. aureus *from milk samples was significantly higher on farms that had fewer than 30 cows, when compared with farms with more than 100 cows (OR = 0.2, 95% CI = 0.11, 0.53, p < 0.005). Also, there was a significantly higher risk of diagnosing *Str. agalactiae *on farms with more than 600 cows (OR = 17.6, 95% CI = 1.2, 259.1, p = 0.034) compared with smaller farms.

### Antimicrobial susceptibility testing

The percentage of *S. aureus *isolates resistant to penicillin and ampicillin was 61.4% and 59.5%, respectively. In addition, CNS showed resistance to penicillin and ampicillin (38.5% and 34.4%), but resistance to erythromycin and lincomycin was also common (14.9% and 17.6%). Six isolates (3.8%) of *S. aureus *and three isolates (3.6%) of CNS were resistant to cephalothin (Table 4).

**Table 4:** Antimicrobial susceptibility of staphylococci isolated from bovine clinical mastitis

	*S. aureus*				CNS			
**Disc content in μg**	**n**	S* (%)	I * (%)	R* (%)	**n**	S* %	I * (%)	R* (%)

Ampicillin10 μg	173	40.5	-	59.5	91	61.5	-	38.5
Penicillin10 μg	174	38.6	-	61.4	93	65.5	-	34.4
Cephalothin 30 μg	160	96.2	-	3.8	84	96.4	-	3.6
Clindamycin 2 μg	169	81.9	0	18.1	91	82.4	0	17.6
Erythromycin15 μg	83	95.2	0	4.8	47	85.1	0	14.9
Tetracycline 30 μg	147	95.9	0	4.1	86	88.4	0	11.6
Trimethoprim/sulfa 1.25/23.75 μg	162	96.6	0	3.4	76	97.4	0	2.6
Gentamycin 10 μg	146	93.2	0	6.8	69	98.6	0	1.4

* Propotion of susceptible (S), intermediate susceptibility (I) and resistant (R) isolates

All streptococci (Table 5) were susceptible to penicillin, ampicillin and cephalothin, except for one isolate of *Str. uberis*. Of the 90 isolates of *Str. dysgalactiae*, 19.8% were classified with intermediate susceptibility and 32.2% with resistance to tetracycline. Of a total of 151 isolates of *Str. uberis*, 7.3% with intermediate susceptibility and 14.3% with resistance to tetracycline were recorded. Among the *E. coli *isolates (Table 6), the highest percentage of isolates showing intermediate susceptibility and resistance were observed with ampicillin, neomycin, streptomycin and tetracycline. *E. coli*. was 98.4% susceptible to enrofloxacin and 100% to cefaperazone.

**Table 5:** Antimicrobial susceptibility of streptococci isolated from bovine clinical mastitis

	*Str. agalactiae*				*Str. dysgalactiae*				*Str. uberis*			
**Disc content in μg**	**n**	S* (%)	I* (%)	R* (%)	**n**	S* (%)	I* (%)	R* (%)	**n**	S* (%)	I* (%)	R* (%)

Ampicillin 10 μg	162	100	-	0	111	100	0	0	265	99.6	0	0.4
Penicillin 10 μg	168	100	-	0	111	100	0	0	267	99.6	0	0.4
Cephalothin 30 μg	143	100	-	0	101	100	0	0	254	99.6	0	0.4
Clindamycin 2 μg	161	91.9	1.9	6.2	115	92.2	0	7.8	273	92	1.4	6.6
Erythromycin 15 μg	77	96.1	2.6	1.3	60	88.3	5	6.7	134	89.6	2.2	8.2
Tetracycline 30 μg	151	78.1	7.3	14.6	90	48.9	18.9	32.2	234	79.9	3.4	19.7
Trimethoprim/sulfa 1.25/23.75 μg	140	93.6	0	6.4	103	99	0	1	223	95.9	0.9	3.2
Gentamycin 10 μg	143	63.6	11.9	24.5	88	88.6	0	11.4	210	71.9	9.5	18.6

* Propotion of susceptible (S), intermediate susceptibility (I) and resistant (R) isolates

**Table 6:** Antimicrobial susceptibility of *E. coli *and *Klebsiella *spp. isolated from bovine clinical mastitis

	*E. coli*				*Klebsiella *spp.			
**Disc content in μg**	**n**	S* (%)	I * (%)	R* (%)	**n**	S* (%)	I* (%)	R* (%)

Ampicillin 10 μg	201	68.7	7.0	24.3	39	15.4	7.7	76.9
Cefaperazone75 μg	137	100	0	0	32	100	0	0
Tetracycline 30 μg	184	77.8	8.7	13.5	39	79.6	10.2	10.2
Trimethoprim/sulfa 1.25/23.75 μg	191	84.3	3.7	12.0	40	97.5	0	2.5
Gentamycin 10 μg	161	94.3	2.5	2.2	40	95.0	0	5.0
Streptomycin 300 μg	154	78.6	5.8	15.6	37	73.0	8.1	18.9
Neomycin 30 μg	155	72.9	20.6	6.5	37	83.8	13.5	2.7
Enrofloxacin 5 μg	185	98.4	0	1.6	37	100	0	0

* Proportion of susceptible (S), intermediate susceptibility (I) and resistant (R) isolates

## Discussion

The results of the present study were based on an analysis of milk samples submitted to an Estonian National Veterinary Laboratory over a three-year period. The laboratory protocols did not change during the study period. Of the samples investigated, 22.3% were bacteriologically negative. Several other studies have also demonstrated bacteriologically negative findings in 17.7-26.5% cases of clinical mastitis [12,25] and as many as 28.7-38.6% of subclinical mastitis [12,26], which is in line with our results. The possible reasons for bacteriologically negative findings in milk samples could be the presence of antibacterial substances in the milk that lead to a decrease in the viability of bacteria in the culture [27], or failures in conventional culture compared with identification of bacteria using the real-time polymerase chain reaction [28].

In the present study, *E. coli *and *Str. uberis *were the pathogens isolated most frequently from clinical mastitis, while *S. aureus*, CNS and *Corynebacterium *spp. caused mainly subclinical mastitis. The same results were shown in an Estonian study ten years ago, where *C. bovis *(47.5%), *S. aureus *(21%) and CNS (15.8%) were the pathogens isolated most commonly from cases of subclinical mastitis [29]. The isolation rate of *Str. agalactiae *was surprisingly high in our study.

We found a strong association between the isolation of *Str. agalactiae *and very large-scale farms. In total, there are 98000 dairy cows in Estonia and the mean herd size is 88 cows [30]. Rapid changes in management style (from tie-stalls to free-stalls) have occurred during the last eight years, which may explain the coexistence of environmental pathogens together with *Str. agalactiae*. Although teat disinfection and dry cow therapy is a common routine on Estonian dairy farms, proper eradication programmes for *Str. agalactiae *have not been employed. In contrast, an increased probability of finding *S. aureus *was correlated with farms with fewer than 30 cows. The average age of cows on small farms was 5.3 years, compared with 4.3 years on farms on which more than 300 cows were kept [30]. The culling policy may be different, and the owners of smaller farms may keep (possibly chronically infected) cows in the herd for a longer period of time.

The disc diffusion method for *in vitro *antimicrobial susceptibility testing was used in this study. This technique is the most widely used method for determination of the susceptibility of animal pathogens, especially in clinical work when it is necessary to determine the correct treatment. The primary disadvantage of using this method when monitoring development of resistance is that outcomes are reported on a qualitative basis (sensitive, intermediate, or resistant), and subtle changes in susceptibility may not be apparent. Therefore any comparison with studies that use other methods of susceptibility testing is not acceptable [31].

Generally in our study, the *in vitro *antimicrobial resistance of the isolates examined from samples of clinical mastitis were high. Isolates of *S. aureus *had an alarming level of resistance to penicillin (61.4%) and ampicillin (59.5%), whereas CNS exhibited a lower degree of resistance to penicillin and ampicillin (38.5%; 34.4%). The reported percentages for penicillin resistant *S. aureus *in cases of clinical mastitis, detected by the disc diffusion method, are 50.4% and 35.4% in the USA [10,32], 63.3% in Turkey [33] and 12% in Northern Germany [34]. In addition, cephalothin resistance among staphylococci was found in our study. Although reports of methicillin-resistant staphylococci causing bovine mastitis are rare, those samples found in our study need further investigation in order to prove or exclude the presence of the *mecA *gene. In the present study, both staphylococci and streptococci showed resistance to erythromycin and lincomycin, but the figures for resistance in annual reports from some other countries show a low prevalence of lincomycin and erythromycin resistance in *S. aureus *and CNS [13,14,35]. Given that *S. aureus *and CNS were the pathogens isolated most frequently from cases of subclinical mastitis, one possible explanation for resistance to several antibiotics may be the collection and submission to the laboratory of milk samples from chronic clinical mastitis (which demonstrate poor treatment efficacy). Therefore, random sampling strategies should be used to provide a good evaluation of antimicrobial susceptibility.

The level of resistance of *E. coli *and *Klebsiella *spp. was high against all tested antimicrobials, except cefaperazone and enrofloxacin. Coliforms are often resistant to more than one antimicrobial [36,37], and the number of multiresistant strains may influence the resistance figures. Coliform bacteria isolated from cases of mastitis may reflect the general situation of resistance in the herd and can be considered more as an indicator of the bacteria present than an indicator of specific pathogens from the udder [36]. All of the bacterial species investigated in the present study showed resistance to tetracycline. A possible explanation for this phenomenon could be that tetracycline has been the class of antimicrobial most widely used for treatment of several infections for many years. In addition, tetracycline has been found in multiresistant patterns with penicillin and streptomycin [33,37].

Statistical data from the Estonian State Medical Agency confirmed [19] that alltogether 209880 single intramammary syringes for lactating cows and 205648 for dry cow therapy were sold in the year 2009. Ampicillin and cloxacillin combinations, cephalosporins with aminoglycosides, and lincomycin with neomycin were the most common choices for the treatment of mastitis in lactating cows. For example, 255 grams of intramammary lincomycin (pure antimicrobial) and 44.2 grams of intramammary cephalosporins per thousand dairy cows were sold for the treatment of clinical mastitis in 2009 [19]. However, only 73.4 grams of penicillin G was used per thousand dairy cows for intramammary treatment of clinical mastitis. The use of broad-spectrum antibiotics and antibiotic combinations may influence the resistance of mastitis pathogens. In addition, bacteriological examination of milk samples before treatment of clinical mastitis is not a common practice in Estonia. According to the available data in Sweden, intramammary and intramuscular penicillin G [38] are used in over 80% of cases for treatment of clinical mastitis, but the prevalence of resistance of *S. aureus *to penicillins is only 7.1% [36]. In Finland, penicillin G and some broad-spectrum β-lactam antibiotics are used in the treatment of clinical mastitis, but the prevalence of resistance in *S. aureus *is only 13% [39]. Bacteriological examination before treatment is common in both countries.

Considering these results, we can assume that the main reason for the occurrence of a high number of resistant strains in Estonian herds is the wide use of broad-spectrum antimicrobials and the long-term presence of infected cows in herds.

## Conclusion

This study showed that the main pathogens that caused clinical mastitis were *Str. uberis *and *E. coli*. Subclinical mastitis was caused mainly by *S. aureus *and CNS. A relatively high number of isolates of *Str. agalactiae *were cultured from both types of case. The number of *S. aureus *and *Str. agalactiae *isolates depended on herd size. Among the bacteria investigated, the prevalence of antimicrobial resistance was extremely high, especially penicillin resistance in *S. aureus *and CNS.

## Competing interests

The authors declare that they have no competing interests.

## Authors' contributions

PK carried out the study, compiled the results and drafted the manuscript, BA participated in data collection and coordinated the laboratory analysis, TO participated in designing the study and statistical analysis of the data, AK performed bacteriological analysis, and KK coordinated the study. All authors were significantly involved in designing the study, interpreting data and composing the manuscript.
